# Surgical and medical management in the treatment of proximal tibial metaphyseal fracture in immature dogs

**DOI:** 10.1371/journal.pone.0268378

**Published:** 2022-06-02

**Authors:** Carly Sullivan, Joshua Zuckerman, Daniel James, Karl Maritato, Emily Morrison, Riccarda Schuenemann, Ron Ben-Amotz

**Affiliations:** 1 Small Animal Surgery, BluePearl Veterinary Partners, Levittown, Pennsylvania, United States of America; 2 Small Animal Surgery, Cape Cod Veterinary Specialists, Buzzards Bay, Massachusetts, United States of America; 3 Small Animal Surgery, Small Animal Specialist Hospital, Sydney, Australia; 4 Small Animal Surgery, MedVet Medical & Cancer Center for Pets, Cincinnati, Ohio, United States of America; 5 Veterinary Radiology, MedVet Chicago, Chicago, Illinois, United States of America; 6 Small Animal Surgery, Small Animal Department, Ear, Nose and Throat Unit, College of Veterinary Medicine, University of Leipzig, Leipzig, Germany; 7 Small Animal Orthopedics, Koret School of Veterinary Medicine, Rehovet, Israel; Indiana University School of Medicine, UNITED STATES

## Abstract

The purpose of this study was to report approaches to surgical and medical management of proximal tibial metaphyseal fractures (PTMF) and short-term case outcome. Medical records of immature dogs with PTMF were reviewed and data were collected including history, signalment and side affected. Data pertaining to surgical and medical management including radiographic evaluation and short-term complications were recorded. Forty-five dogs with a total of 47 PTMF identified and treated between 2007–2019 were included in this study. Six cases were managed with external coaptation alone. Forty-one cases were treated surgically with constructs including K-wires in different configurations, bone plate and screws, and external skeletal fixation. Of the cases managed conservatively, 4 developed complications, including bandage sores, diffuse osteopenia of the tarsus/metatarsus, and angular limb deformities. Surgical complications including pin migration necessitating removal, osteopenia, and screw placement in the proximal tibial growth plate or into the stifle joint were found in 16 cases. PTMF treated with surgery had a subjectively more predictable outcome compared to those treated with external coaptation alone. Conservative management may result in complications including development of excessive tibial plateau angle (TPA) as well as distal tibial valgus.

## Introduction

Tibial fractures account for approximately 20% of all long bone fractures in companion animals, making them the third most commonly occurring fracture [1, 2]. Fractures involving the proximal tibial metaphysis are relatively uncommon, and are reported to comprise 3.7% of all tibial fractures [3]. Other fractures of the proximal tibia include tibial tuberosity avulsion fractures, Salter Harris Type II fractures, and combined tibial tuberosity avulsion and proximal physeal fractures [4, 5].

**Fig 1:**
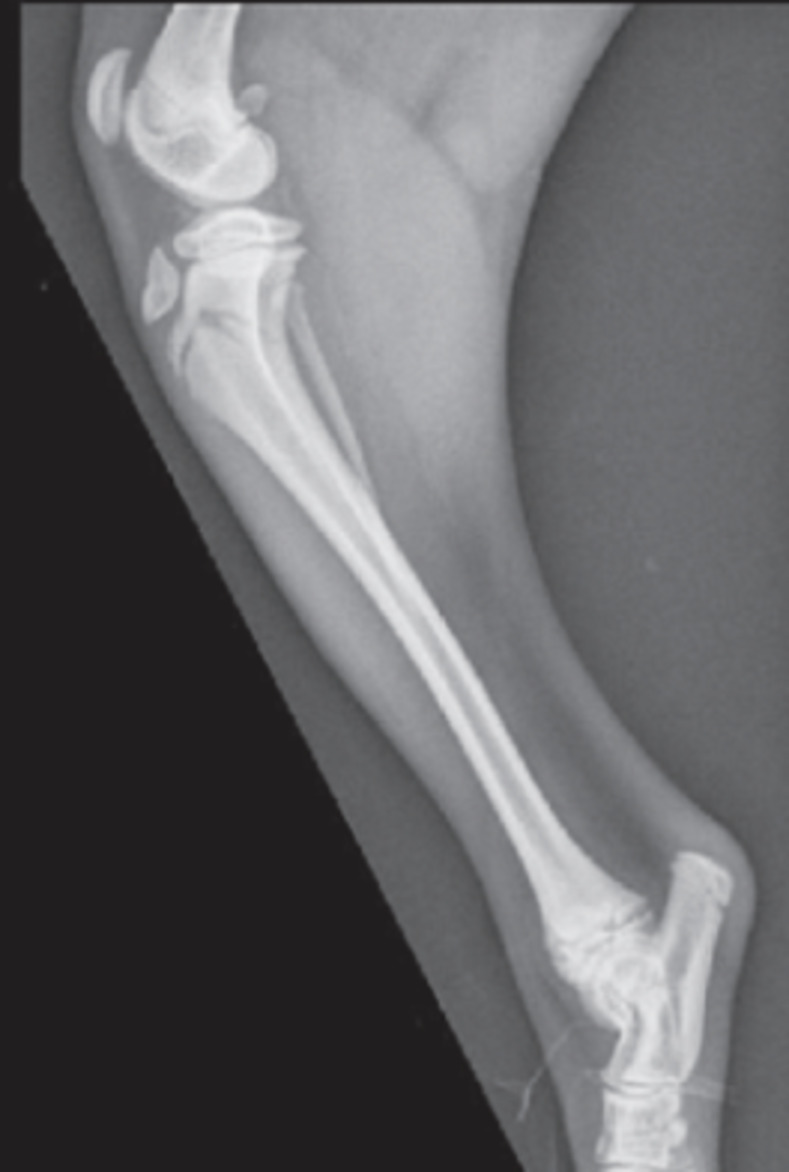
Characteristic curvilinear configuration of the proximal tibia seen with PTMF.

Until recently, it had been suggested that fractures of the proximal tibial metaphysis were exclusive to mature animals secondary to severe trauma [6–8]. However, Deahl et al. reported the occurrence of proximal tibial metaphyseal fractures (PTMF) in juvenile dogs (mean age 4.6 months) following minimal trauma [9]. Most commonly, PTMF manifest in a characteristic curvilinear configuration (Fig 1). The authors of that study suggested that the transition from diaphyseal to metaphyseal bone and the immature or transition zone of the metaphysis play a role in the development of this fracture configuration [9].

**Fig 2:**
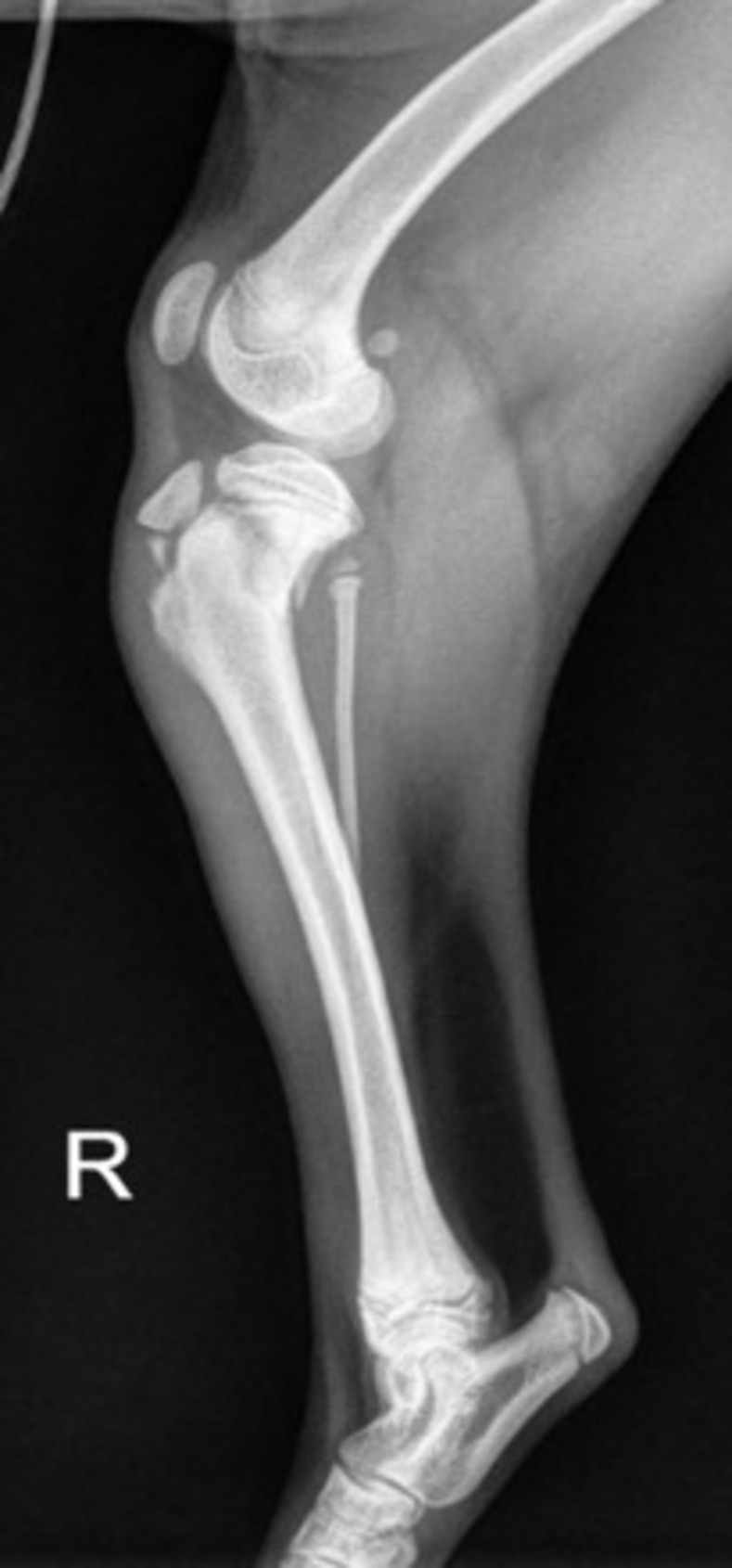
Mediolateral radiograph of a PTMF demonstrating cranial displacement of the distal fragment and caudal tipping of the proximal tibia resulting in an increased tibial plateau angle.

**Fig 3:**
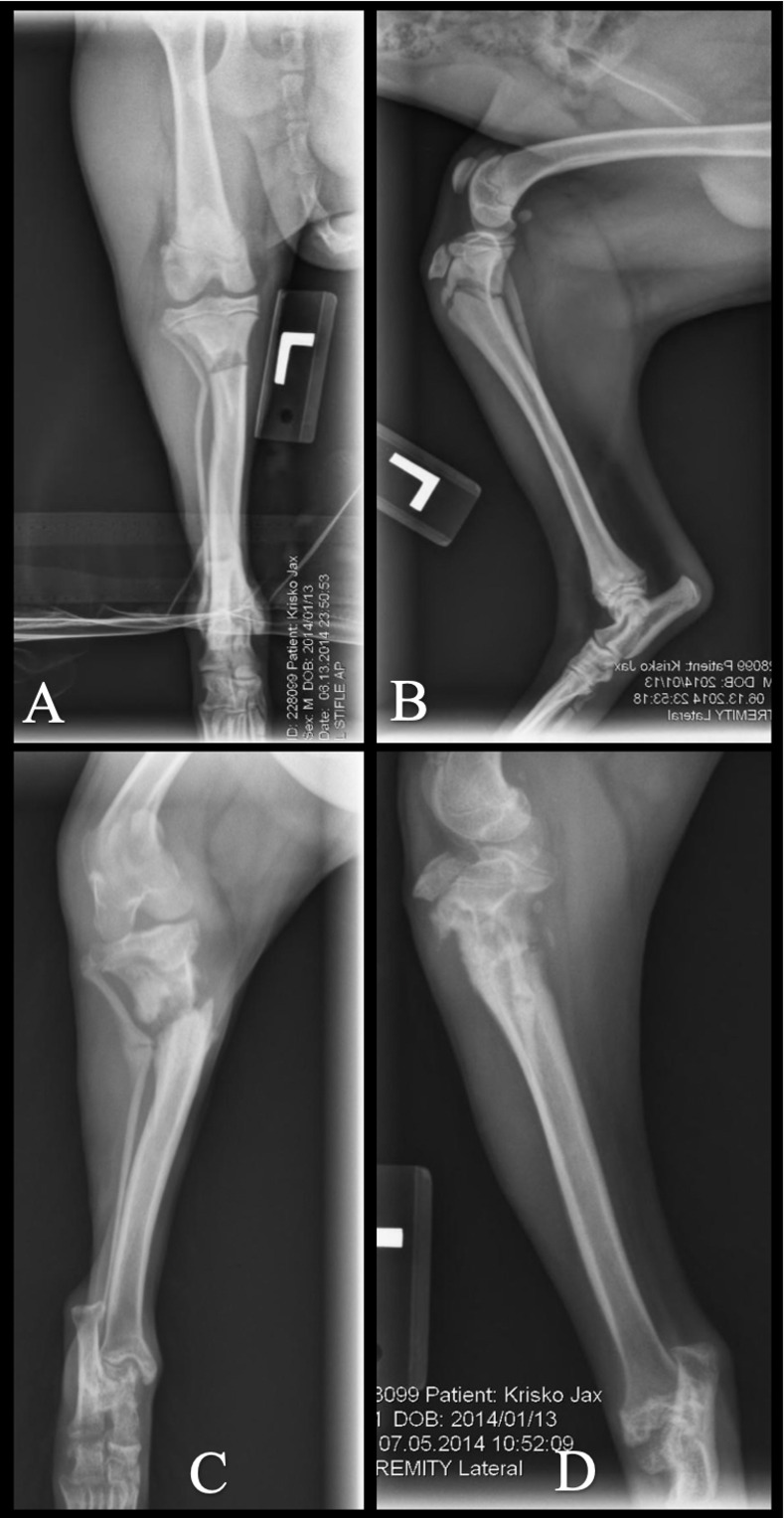
Case 34 managed with a splint bandage alone. A and B: Mediolateral and craniocaudal views at time of injury. C and D: Mediolateral and craniocaudal views 3 weeks post injury demonstrating valgus deviation of the distal tibia. Ultimately development of genu varum, medially luxating patella, tibial tuberosity avulsion fracture, patella alta, tarsal osteopenia, and fibular malunion led to an amputation.

In most reported proximal metaphyseal tibia fractures, craniomedial displacement of the distal tibia fragment relative to the proximal fragment occurs. This results in caudolateral angulation of the distal limb. The cranial displacement of the distal fragment and caudal tipping of the proximal tibia increases the risk for development of a steep tibial plateau angle and therefore increased strain on the cranial cruciate ligament as it heals (Fig 2) [10]. In the frontal plane, these fractures may also result in valgus angulation in the distal fragment (Fig 3C).

Clinical outcomes for dogs following management of PTMF have not been reported in the literature. Treatment options for stabilization of these fractures are influenced by multiple factors, including patient age, the presence of open physes, degree of fragment displacement, availability of proximal metaphyseal bone stock, and cost to the client.

The objective of this study was to report on surgical and conservative approaches to the management of PTMF, including complications and short-term outcome.

### Study design

Medical records of dogs that presented with a PTMF between the years of 2015–2020 were collected from several veterinary referral hospitals. Data retrieved from the medical records included breed, weight, side affected, age at the time of presentation, gender, sterilization status at the time of the injury, details of the inciting trauma, displacement, presence of concurrent fibular fracture, stabilization technique (external coaptation vs. surgical fixation) and recorded complications.

## Results

Forty-seven fractures occurring in 45 dogs were available for review (Table 1).

**Table 1:** Description of all cases.

Case Number	Age at time of injury (weeks)	Gender	Breed	Side	Type of Injury	Displacement	Fibula fracture?	Management	External coaptation	Complications?	Outcome
1	17	F	Prague Ratter Dog	Left	Fell from owner’s arms	Craniomedial	Yes	1.2 mm and 0.8 mm IM pins and another 0.8 mm cross pin, TBW with PDS	None	None	Recovered well
2	23	M	Chihuahua	Not Specified	Fell from owner’s arms	Cranial	Yes	IM pin and external fixator type I	Only around fixator	None	Recovered well, fixator removed
3	14	M	Chihuahua	Right	Minor trauma during unaccompanied exercise outside—presented 10 days after injury	Cranial	No	3 k-wires and PDS TBW	1 week SPB	None	Recovered well
4	13	F	Mix	Left	Jumped off roof	Cranial	Yes	3 1.2 mm k-wires and PDS TBW	MRJ bandage recommended but patient lost to follow-up	Lost to follow-up	Lost to follow-up
5	22	M	Miniature Poodle	Not Specified	Jumped out of owner’s arms 3.5 weeks prior to presentation	Cranial	Yes	Not specified	Not specified	Not specified	No surgery—mostly healed at time of consultation but surgery was recommended
6	15	M	Terrier Mix	Right	Dropped from low height	Craniolateral	Yes	ORIF with non-locking T-plate	None	Valgus deformity—screw impingement on lateral portion of proximal tibial physis	?
7	13	M	Boston Terrier	Left	Dropped from low height	No preoperative radiographs to evaluate	Yes	ORIF with non-locking T-plate	None	None	Recovered well
8	18	M	Toy Poodle	Left	Dropped from low height	Craniomedial	Yes	ORIF with locking T-plate	None	Valgus deviation at the fracture site	?
9	10	FS	Poodle x CKCS	Left	Fell while running	Cranial	No	ORIF with crossed k-wire fixation	None	None	Recovered well
10	11	F	Chihuahua	Right	Dog fight with history of being dropped 1 week prior to fight	Medial	Yes	ORIF with crossed k-wires and non-locking L-plate fixation	None	Discharging sinus without lameness 3 months later resulted in explantation	Recovered well after explantation
11	16	F	Pomeranian	Left	Unknown trauma	Craniomedial	Yes	ORIF with L locking plate	None	Most proximal screws in proximolateral tibial physis	?
12	14	F	Toy Poodle	Left	Dropped from low height	Cranial	No	ORIF with k-wire fixation	None	Explantation after fracture healed	Recovered well after explantation
13	13	F	CKCS	Right	Fell from couch	Craniolateral	Yes	ORIF with non-locking T-plate	Not specified	None noted	Recovered well
14	18	F	French Bulldog	Not Specified	Unknown trauma	Unable to assess on radiographs	No	ORIF with non-locking T-plate	Not specified	External rotation and valgus on radiographs but ambulates well	Recovered well
15	20	F	Toy Poodle	Left	Jumped from sleeper	None	Yes	Mini non-locking T-plate after splinted by pDVM for 2 weeks	None	Valgus deforming but functional, proximal screw into the joint and through proximolateral physis	?
16	Not specified	Not specified	Not specified	Left	Not specified	Cranial	Yes	ORIF with k-wire fixation	None	Not specified	Recovered well
17	24	MN	Not specified	Left	Jumped off bed	None	No	2.7 mm locking T plate	Not specified	Disuse osteopenia of tarsus, proximal screw into joint, physeal violation with screw with increased TPA	Recovered well
18	20	M	Rat Terrier	Right	Unknown trauma at home	None	Yes	Cross pins	None	None	Recovered well
19	20	F	Sheltie	Left	Fell off couch	Only PO radiographs available	No	Cross pins	None	None	Recovered well
20	13	F	Boston Terrier	Left	Unknown trauma at home	Unable to assess on radiographs	No	Cross pins	None	None	Recovered well
21		M	Chihuahua Mix	Left	Unknown trauma at home	Only PO radiographs available	Yes	Cross pins	None	Pin migration	Recovered well after explantation
22	18	F	Boston Terrier	Left	Unknown trauma outside in yard alone	None	No	ORIF with locking T-plate	None	None	Recovered well
23	19	M	French Bulldog	Right	Fell down the stairs	Only PO radiographs available	No	IM pin with antirotational pin	None	None	Recovered well
24	16	F	French Bulldog	Right	Fell from steps	Only PO radiographs available	No	PAX T-plate	None	None	Recovered well
25	24	M	Yorkie	Right	Fell from arms	Caudal	Yes	3 cross pins	None	None	Recovered well
26	24	F	Chihuahua	Right	Unknown trauma at home	Caudal	No	3 cross pins	None	None	Recovered well
27	17	F	Toy Poodle	Right	Fell off deck	Only PO radiographs available	Yes	PAX T-plate	None	Infection post-operatively	Recovered well after treatment for infection
28	39	MN	Maltese	Right	Fell from arms	Caudolateral	Yes	3 cross pins	None	Pin migration	Recovered well after explantation
29	20	FS	Yorkie	Left	Unknown trauma at home	Medial	Yes	3 cross pins	None	Pin migration	Recovered well after explantation
30	20	FS	French Bulldog	Right	Unknown trauma at home	Only PO radiographs available	Yes	3 cross pins	None	None	Recovered well
31	19	F	French Bulldog	Right	Fell off couch	Cranial	Yes	3 cross pins	None	None	Recovered well
32	18	F	Boston Terrier	Right	Fell from bed	Craniomedial	Yes	IM pin	Cranial splint for 3 weeks, SPB for 1 week	Patella riding medially but unable to luxate	Occasionally lame at home
33	24	M	French Bulldog	Right	Fell from couch	Craniomedial	Yes	2 cross pins, 4 pins including 1 down tibial shaft, MPL correction	2 weeks for 1st surgery (pins in joint), 4 weeks for second surgery	Fractured 2 weeks post first surgery with 2 k-wires—repeated surgery with 4 pins at divergent angles and bandaged for 4 weeks. 6 months post 1st surgery, grade 4/4 MPL corrected, 8 months post 1st surgery, pin removal for TTT	Recovered well after last procedure
34	20	M	Poodle/Terrier Mix	Left	Jumped out of stopped car	Cranial	Yes	Cranial splint for 6 weeks	Cranial splint for 6 weeks	Non-healing malunion developing angular limb deformity (genu varum), MPL, tibia tuberosity avulsion, patella alta, tarsal osteopenia, fibular malunion	Left mid-femoral amputation
35	20	M	Yorkie	Left	Fell down the stairs	Cranial	Yes	2 0.045 k-wires	Cranial splint for 2 weeks	Pin migration	Recovered well after explantation
36	24	F	Terrier Mix	Left	Stepped on by owner	Cranial	Yes	3 0.045 k-wires	None	None	Recovered well
37a	18	M	Chihuahua	Bilateral—Right	Jump off couch	Only PO radiographs available	Yes	2 0.045 k-wires	Cranial splint for 3 weeks, SPB for 1 weeks	Diffuse osteopenia of the tarsus and metatarsus	Recovered well once external coaptation was removed
37b	18	M	Chihuahua	Bilateral—Left	Jump off couch	Only PO radiographs available	Yes	2 0.045 k-wires	Cranial splint for 3 weeks, SPB for 1 weeks	Diffuse osteopenia of the tarsus and metatarsus	Recovered well once external coaptation was removed
38	16	M	Corgi	Right	Jump off bench	Caudal	Yes	2 1/16 pins	SPB overnight	None reported	Lost to follow-up
39	16	F	Chihuahua	Right	Fell from bed	None	No	2 0.045 k-wires	SPB for 1 week	None reported	Lost to follow-up
40a	20	M	Chihuahua Mix	Bilateral—Left	Jump from owners’ arms	Cranial	No	Lateral splint for 2 weeks, SPB for 2 weeks	Lateral splint for 2 weeks, SPB for 2 weeks	Internal tibial rotation, diffuse osteopenia of tarsus and metatarsus, changes to the metatarsus	Grade 1-2/4 MPL
40b	20	M	Chihuahua Mix	Bilateral—Right	Jump from owners’ arms	Craniolateral	Yes	Lateral splint for 3 weeks, SPB for 3 weeks	Lateral splint for 3 weeks, SPB for 3 weeks	Internal tibial rotation, diffuse osteopenia of tarsus and metatarsus, changes to the metatarsus	Patella rides on medial trochlear ridge but unable to luxate
41	16	MN	Chihuahua Mix	Left	Jumped from owners’ arms	Caudal	Yes	0.045 k-wires, 2.0 DCP plate	Splint for 2 weeks, SPB for 2 weeks	Screw through proximolateral tibia physis on immediate PO radiographs but not at recheck	Recovered well
42	24	F	Chihuahua	Not specified	Not specified	Only post treatment radiographs available	Unsure	Splint for 4 weeks	Splint for 4 weeks	None	Recovered well
43	Not specified	Not specified	Not specified	Left	Not specified	None	Yes	3 cross pins	None	None	Recovered well
44	16	M	Terrier Mix	Left	Jumped from bed	Caudomedial	Yes	TPLO plate	SPB for 1 week	None	Recovered well
45	13	FS	Minatare Poodle	Left	Jumped from bed	Craniomedial	No	Splint for 4w - changed weekly	Splint for 4w - changed weekly	Mild skin sores, valgus, disuse osteopenia	Recovered well

IM–intramedullary

TBW–tension band wire

PDS–polydioxanone

K-wire–Kirschner wire

SPB–soft padded bandage

MRJ–modified Robert Jones

ORIF–open reduction, internal fixation

MPL–medial patellar luxation

TTT–tibial tuberosity transposition

PO—postoperative

TPLO–tibial plateau leveling osteotomy

### Signalment

Mean age at the time of the injury was 18.5 weeks (range from 10–39 weeks). Twenty-three cases were female dogs and 20 cases were male dogs. Two cases did not have this data available for review. Sixteen of the cases were intact females and 4 were spayed females. Seventeen cases were intact males and 3 cases were neutered males. In 5 dogs, sterilization status was not recorded. Represented breeds included mixed breed (9), Chihuahua (7), French Bulldog (6), Miniature Poodle (6), Boston Terrier (4), Yorkshire Terrier (3), Corgi (1), Cavalier King Charles Spaniel (1), Maltese (1), Prague Ratter (1), Pomeranian (1), Rat Terrier (1), and Shetland Sheepdog (1). Breed was not recorded in 3 cases. Mean weight was 3.9 kg (range from 0.8–9.9 kg).

### Laterality

The right tibia was affected in 17 cases and the left tibia was affected in 22 cases. Two cases were bilaterally affected. Four cases were lacking information regarding laterality.

### Fracture causes

In all but three cases, the reported cause of the fracture was a fall or jump from a low height, which was consistent with reported causes described in Deahl et al. [9] The medical records for the remaining three cases did not contain information regarding inciting cause.

### Concurrent fibular fracture

Concurrent fibular fracture occurred in 33 of the 46 cases with mediolateral and craniocaudal radiographs available. Of those managed with external coaptation alone, 3 cases had a concurrent fibular fracture and 2 had intact fibulae. Access to only one radiographic view of one medically managed case made it challenging to determine whether a fibular fracture was present. Of the cases managed surgically, 30 sustained fibular fractures and 12 did not have a fibular fracture.

### Fracture treatment

Of the 47 fractures available for review, 6 were treated non-surgically with external coaptation consisting of a bandage and splint for a mean period of 5 weeks (Table 1).

**Fig 4:**
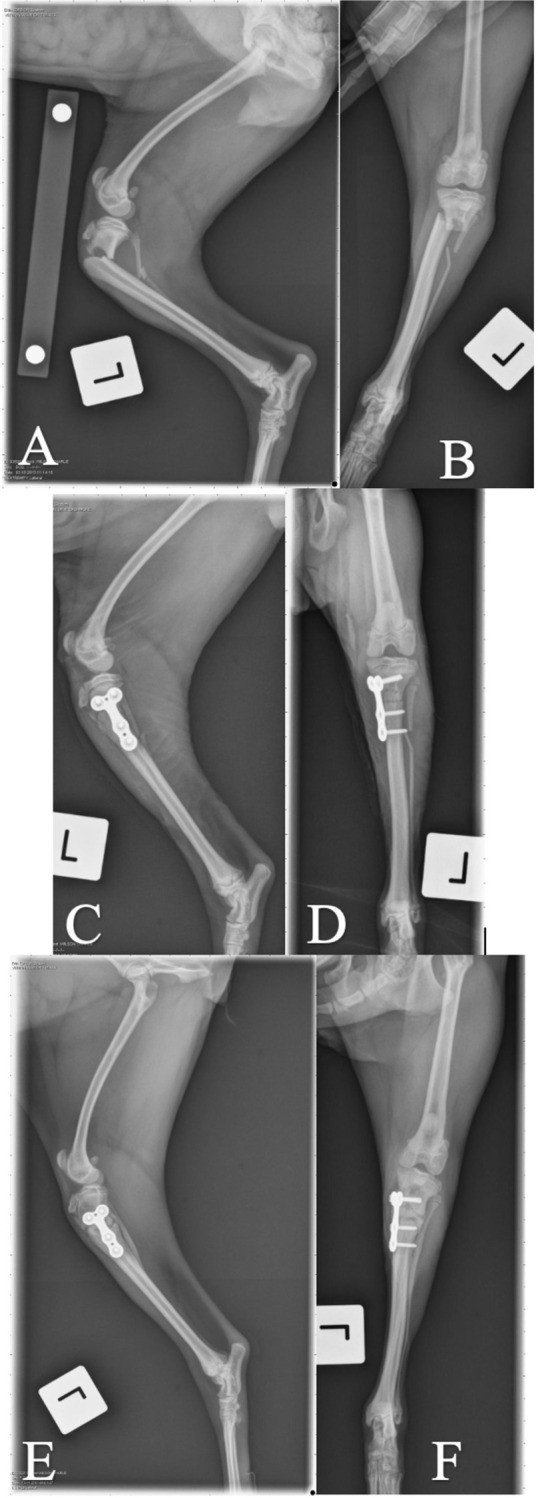
Case 8 managed with locking T-plate for stabilization of PTMF. A and B: Preoperative mediolateral and craniocaudal views. C and D: Immediate postoperative mediolateral and craniocaudal views. E and F: 6 weeks postoperative mediolateral and craniocaudal views.

**Fig 5:**
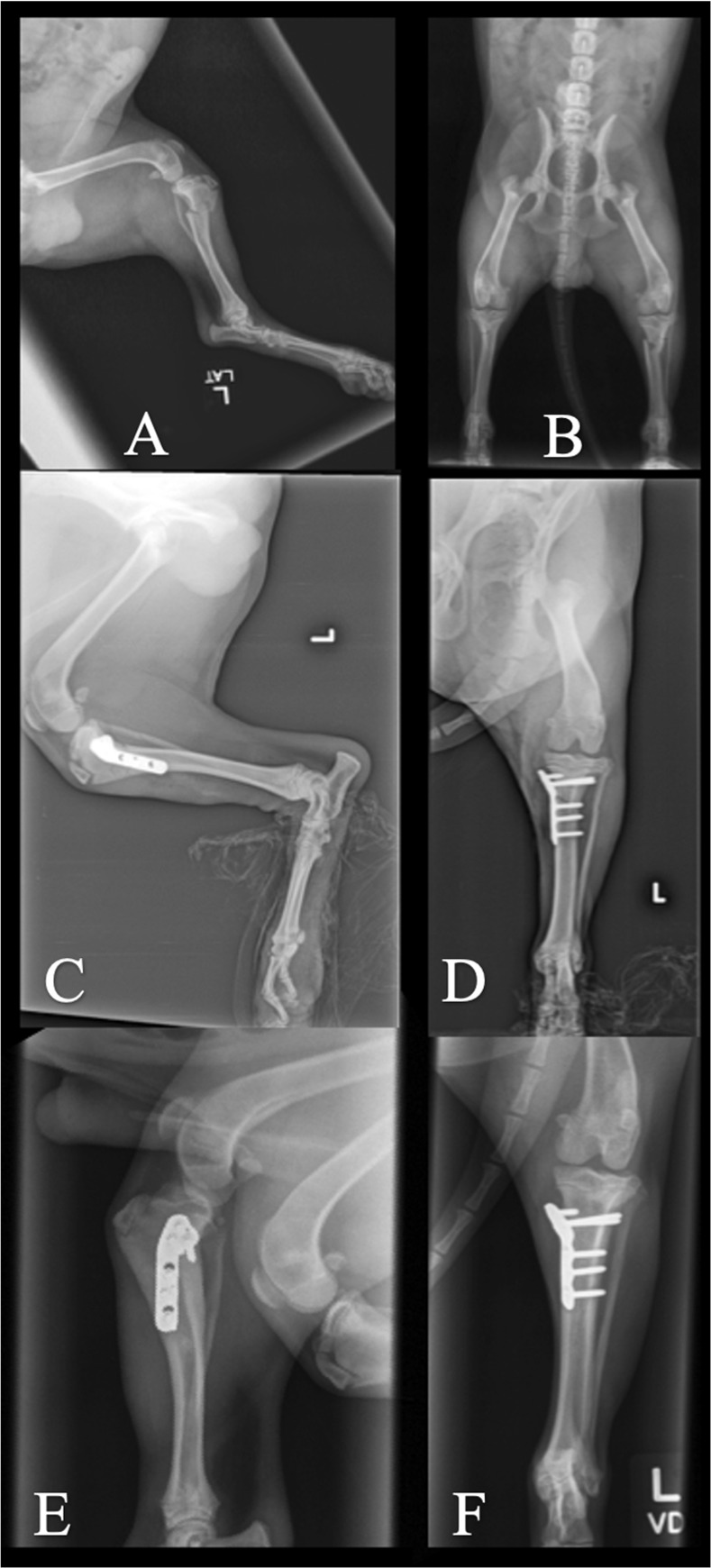
Case 47 managed using a locking TPLO plate. A and B: Mediolateral and craniocaudal views. C and D: Immediate postoperative mediolateral and craniocaudal views. E and F: 4 weeks postoperative mediolateral and craniocaudal views.

Forty-one fractures were treated surgically. Fracture stabilization using K-wires was performed in 26 cases, 23 of which were repaired with a cross-pinning technique, and 3 of which were repaired with pins and a tension band wire. A soft padded bandage or cranial splint was applied in 9 of these cases for a mean of 17.2 days postoperatively. In 14 cases, stabilization was performed using a bone plate and screws (Table 1). Plates applied included a non-locking T-plate (5), locking T-plate (7) (Fig 4), locking L-plate (1), non-locking L-plate (1), locking TPLO plate (1) (Fig 5), and a dynamic compression plate (1). Two of the plated cases were treated with either a soft padded bandage or lateral splint for a mean of 17.5 days postoperatively.

**Fig 6:**
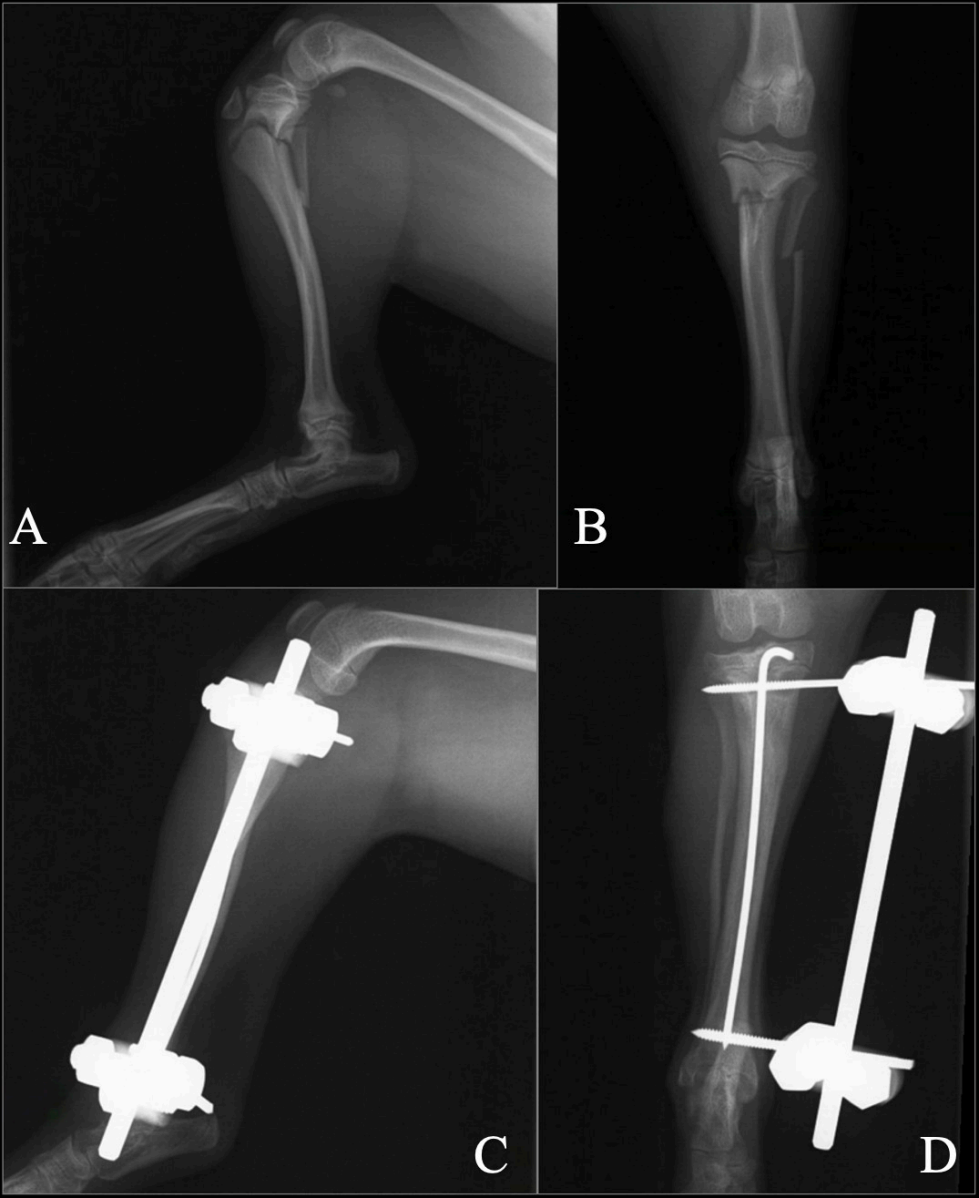
Intramedullary pin and modified type 1a external fixator (Case 2). A and B: Preoperative mediolateral and craniocaudal views. C and D: 8 weeks postoperative radiographs: mediolateral and craniocaudal views.

One case was treated with an intramedullary pin and modified type 1a external fixator for 8 weeks (Fig 6).

### Complications

**Fig 7:**
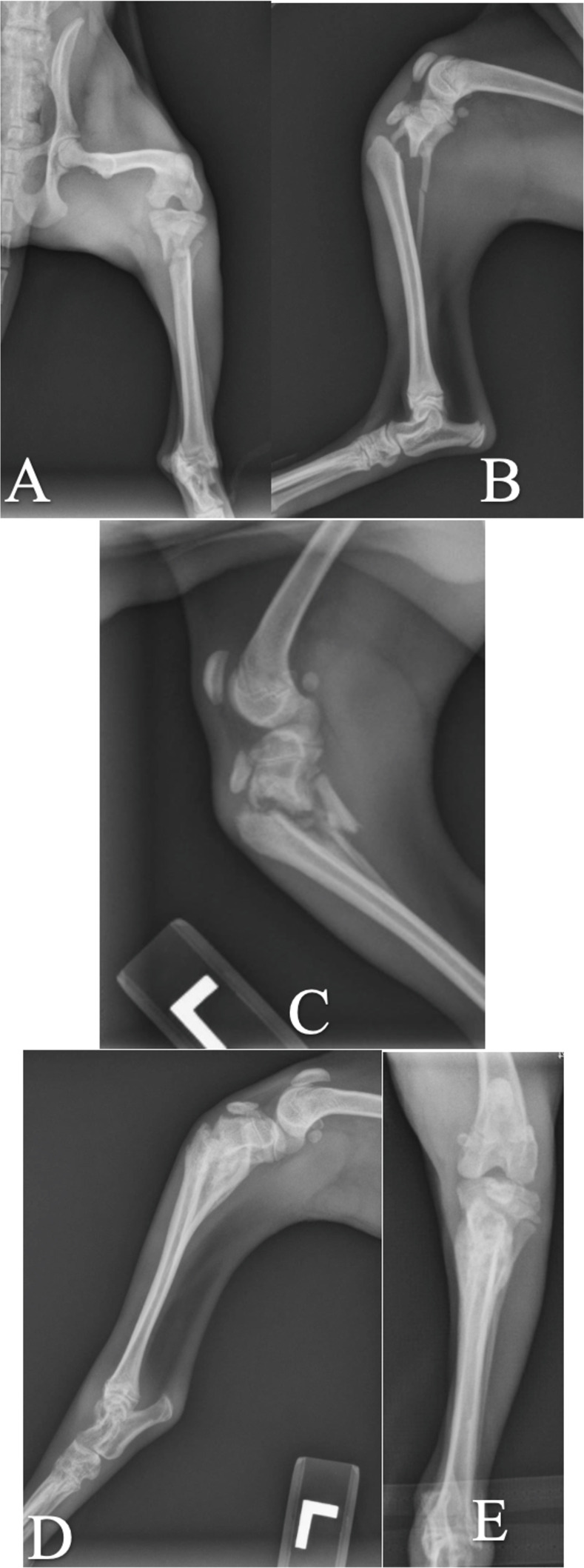
Case 40a managed with a splint bandage alone. A and B: Mediolateral and craniocaudal views at time of injury. C: Mediolateral view 2 weeks post injury. D and E: Mediolateral and craniocaudal views 6 weeks post injury demonstrating excessive TPA that can result from treatment with external coaptation alone.

Of the 6 cases managed with external coaptation alone, 4 developed complications. Following the development of genu varum, medial patellar luxation, tibial tuberosity avulsion fracture, patella alta, tarsal osteopenia, and fibular malunion after 6 weeks of management in a cranial splint (Fig 3), one patient ultimately underwent amputation of the left pelvic limb (case 34). One bilaterally affected case developed internal tibial rotation and excessive TPA with bilateral medial patellar luxation and diffuse osteopenia of the tarsus and metatarsus. The patellar luxations ultimately required surgical correction (case 40, Fig 7). The remaining case developed bandage sores and disuse osteopenia that resolved following bandage removal (case 45).

**Fig 8:**
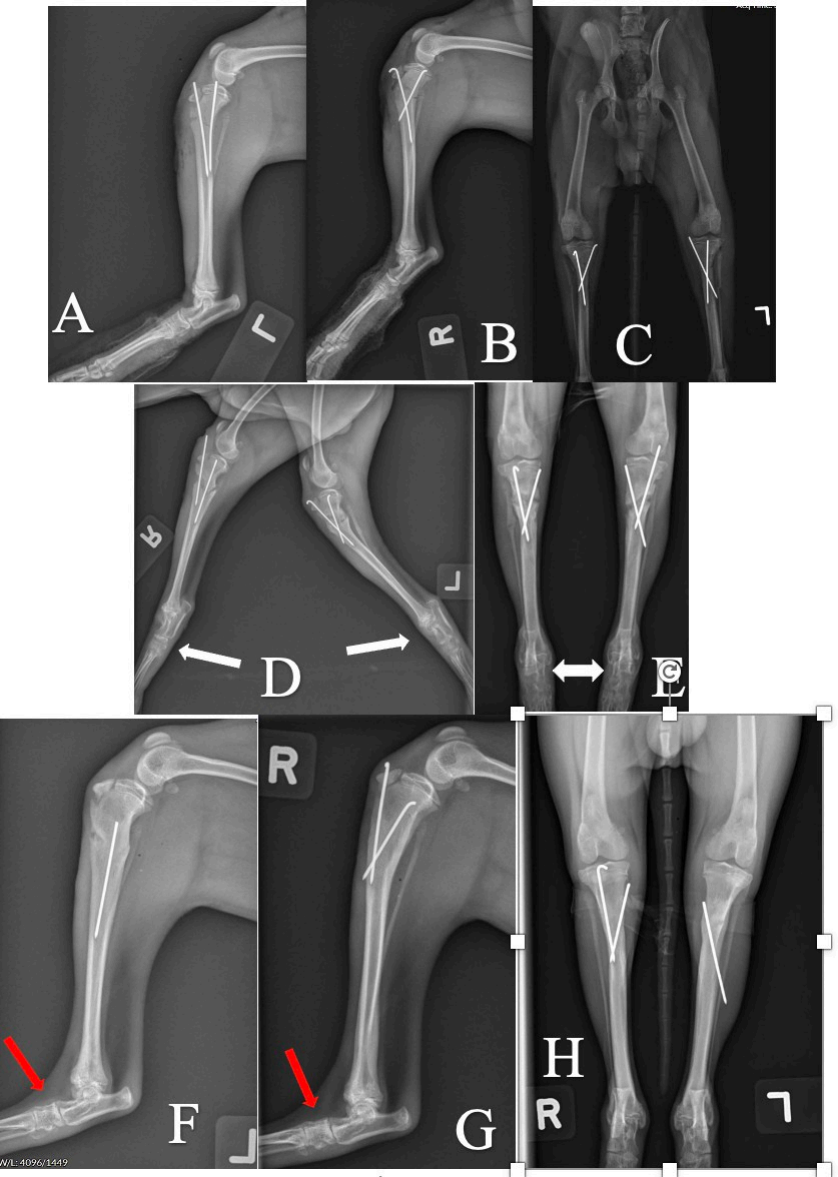
Development of diffuse osteopenia of the tarsus and metatarsus after a bandage was placed for 4 weeks postoperatively (case 37). A, B and C: Bilateral mediolateral and craniocaudal views immediately postoperative. D and E: Mediolateral and craniocaudal views 4 weeks postoperative. There is progressive healing of the proximal tibial fractures. Severe osteopenia is present affecting the tarsal cuboidal bones and proximal metatarsal bones (white arrows). F, G and H: Mediolateral and craniocaudal views 8 weeks postoperative. The proximal tibial fractures have healed appropriately. Mild to moderate osteopenia of distal limbs, but improved compared to radiographs at 4 weeks after surgery (white arrows).

Complications were recorded in 9 cases that underwent surgical repair using pins or pins and tension band wire. Pin migration or breakage was the most common complication and occurred in 6 cases (cases 12, 21, 28, 29, 33, and 35) all of which required a second surgery to either replace or remove the displaced implants. In one case (case 37a and b), external coaptation was used as repair augmentation (3 weeks of a cranial splint followed by 1 week of modified Robert Jones bandage). This resulted in development of diffuse osteopenia of the tarsus and metatarsus, which improved following bandage removal (Fig 8). The remaining pin construct case with a complication was continued to exhibit an intermittent lameness at the time of the last follow-up 8 weeks after surgery. In this case it was noted that the patella rode along the medial trochlear ridge but could not be luxated (case 32).

**Fig 9:**
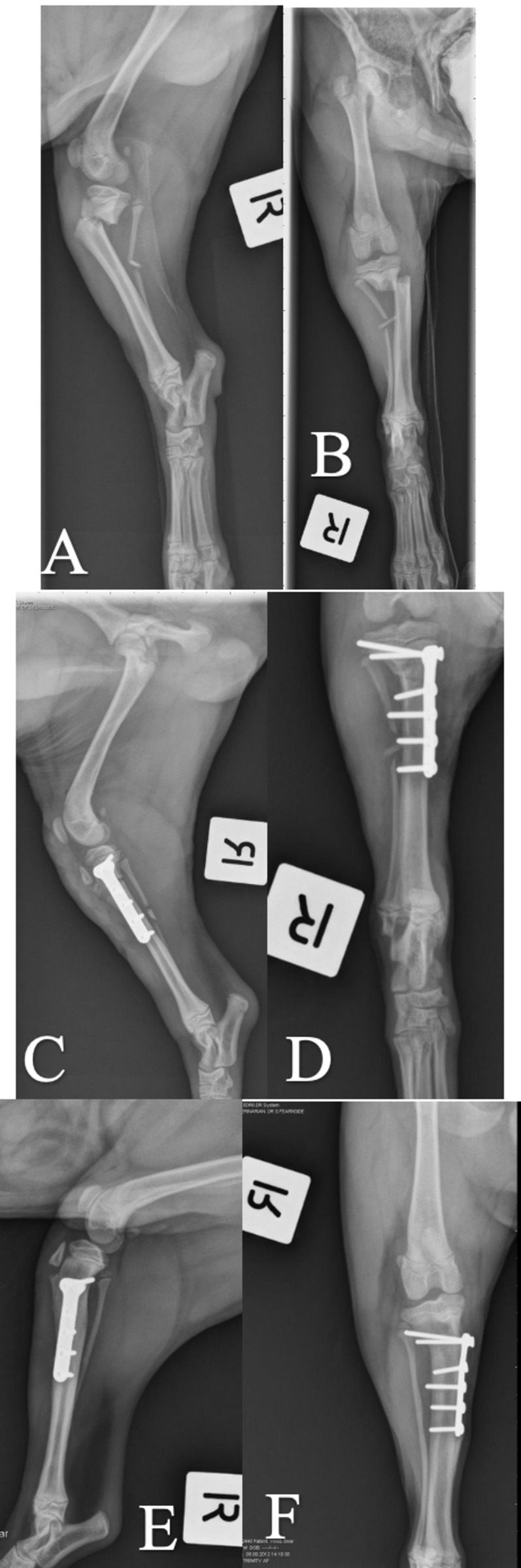
Inadvertent placement of the most proximal screw through the proximal tibial physis resulting in valgus deviation of the proximal tibia (Case 6). A and B: Mediolateral and craniocaudal views at time of fracture diagnosis. C and D: Immediate postoperative mediolateral and craniocaudal views. E and F: 6 weeks postoperative mediolateral and craniocaudal views.

**Fig 10:**
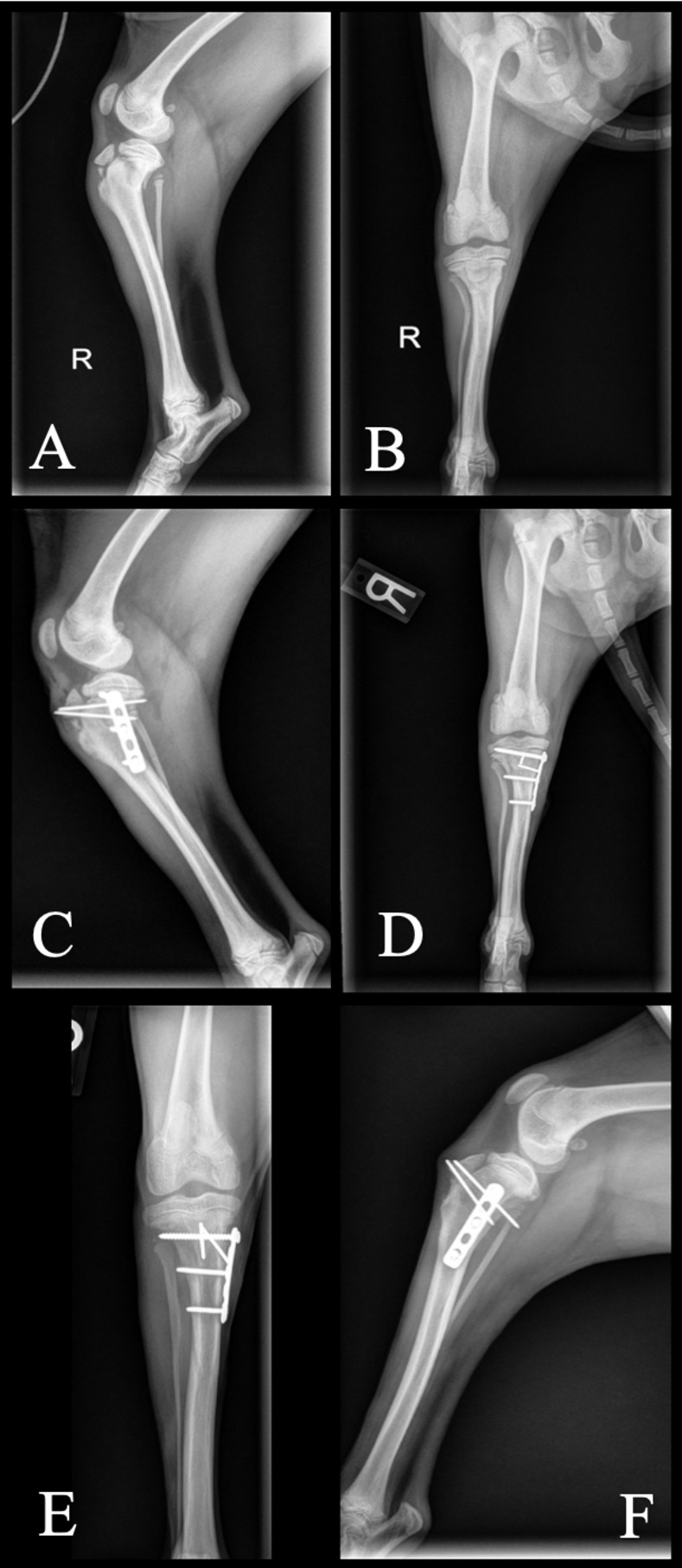
Case 41 in which the proximal screw violated the proximal tibial physis immediately postoperatively on radiographs, but was not within the proximal tibial physis at radiographic evaluation 4 weeks after surgery. A and B: Mediolateral and craniocaudal views at time of injury. C and D: Immediate postoperative mediolateral and craniocaudal views. E and F: 4 weeks postoperative radiographs mediolateral and craniocaudal views.

**Fig 11:**
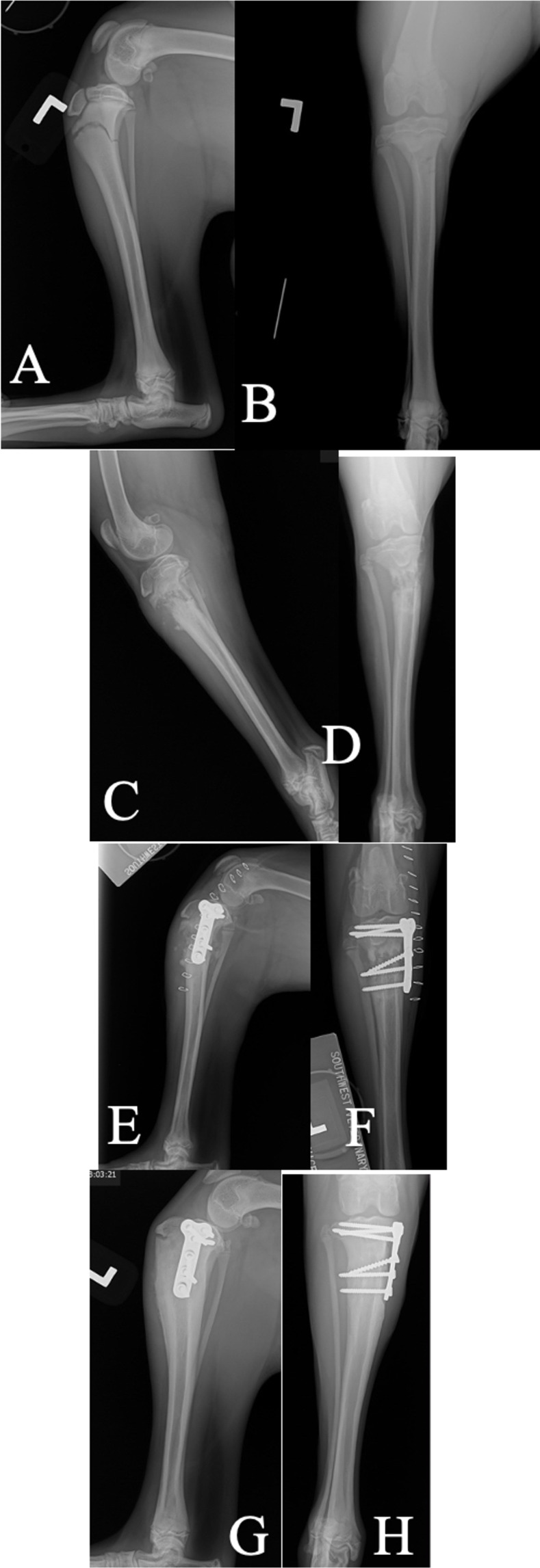
Inadvertent place of the most proximal screw through the proximal tibial physis and into stifle joint after the case was first medically managed with a splint bandage (Case 17). A and B: Mediolateral and craniocaudal views performed 1 day after the initial injury: C and D: Mediolateral and craniocaudal views performed 3 weeks post splint placement. There was concern for collapsing of the lateral tibial cortex resulting in increased tibial angulation. The patient was still grade 4/4 lame on exam, so surgical stabilization was elected. E and F: Mediolateral and craniocaudal views immediately postoperatively showing screw placement into the joint. G and H: Mediolateral and craniocaudal views performed 6 weeks postoperatively with screw placement still within the joint, but patient was not lame or painful on exam.

Seven cases that underwent internal fixation using a plate and screws developed complications. The most proximal screw passed through the proximal tibial physis and was left in place (case 6 (Fig 9), 11, and 41 (Fig 10). This resulted in development of a valgus deformity at the fracture site in 2 cases (case 6 and 11). There was no apparent dysfunction of the limb during follow-up. In two cases, a screw violated the proximal tibial physis and penetrated the stifle joint (case 15 and 17, Fig 11). One case was lost to follow up and the other recovered well without any reported lameness. The remaining two cases treated with plate and screw fixation developed surgical site infections that resolved with either implant removal 12 weeks after surgery or oral antibiotic therapy (case 10 and 27, respectively).

## Discussion

Both surgical and conservative approaches to the management of proximal tibial metaphyseal fractures have been described. Clinically, patients treated with surgical stabilization appeared to have superior outcomes compared to those treated with external coaptation alone. However, the small sample size of the present study precludes the demonstration of statistical significance.

Despite the small sample site, the authors believe there is evidence to suggest that surgical stabilization of these cases achieves better clinical outcome with a lower risk of severe complications compared to use of external coaptation alone. Fracture of the proximal tibial metaphysis typically occurs in juvenile dogs [6], and the use of external coaptation during periods of sustained growth may result in complications including angular limb deformity, muscle contracture, and/or disuse osteopenia [11]. Additionally, bandages may also contribute to the development of sores, swelling, and dermatitis [12]. One patient in this study required amputation of the affected limb as a direct result of complications arising from external coaptation. Given the potential for significant complications, the authors believe external coaptation alone should only be considered for cases in which limited financial resources make surgical correction impossible.

Of the fractures that underwent primary surgical repair, 26 were stabilized using a pin construct, 14 were stabilized using a bone plate and screws and one was stabilized with a type 1a external fixator. Regardless of stabilization method, all surgical cases had a more predictable outcome when compared to the cases managed with external coaptation alone.

Internal fixation constructs using pins included cross pinning, multiple diverging K-wires and intramedullary pin placement. The most common complication encountered in these cases was pin migration, often necessitating removal following documentation of adequate fracture healing. Three previous studies on fracture pinning showed a variable pin removal rate after reduction of physeal fractures. Boekhout-Ta et al. reported a pin migration rate of 4% and elective pin removal due to irritation was performed in 41% of cases in this study [13]. In 2004, it was reported that no pins migrated or were removed in 7 young dogs undergoing open reduction and internal fixation of proximal tibial fractures [14]. Another study in 1989 evaluated blind pinning of the tibia and femur in dogs. There was a 71% pin removal rate reported in 7 physeal fractures [15]. At this time, more information is needed regarding pin migration rate and removal for cases of PTMF, although removal is considered standard of care if migration or irritation occurs.

Subjectively, we appreciated an increased difficulty associated with reduction of the fracture site during surgery when pins were used. Interestingly, even in dogs that underwent primary repair with pins, bandages appeared to increase the risk of complications, though the significance of this finding could not be demonstrated due to low case numbers. However, the use of external coaptation following repair of PTMF with pins should be approached cautiously. It is recommended to closely follow up on cases that were repaired with pins both clinically and radiographically in order to address any complications such as pin migration and soft tissue mobility.

The most common complication encountered in the cases managed with a bone plate and screws was inadvertent placement of the most proximal screw through the proximal tibial physis. This resulted in valgus deviation of the proximal tibia in 2 cases (Fig 9), but did not affect the clinical outcome of these patients in the short-term. Long-term follow up would be necessary to fully determine whether this change is clinically significant. One case with physeal violation continued grow normally and did not develop distal tibial valgus (case 41, Fig 10). Kennon et al. described that limited central transphyseal bridging that occurs after physeal injury can be associated with continued normal hydrostatic bone growth to overcome physeal violation, which may explain the outcome of this case [16]. In two cases, the proximal screw violated the proximal tibial physis and penetrated the stifle joint. It is likely that the use of intraoperative fluoroscopy would greatly diminish the risk of this complication. There is no indication in the medical records as to why these cases were not immediately re-operated to achieve appropriate screw placement. Possible reasons for this may be due to limited bone stock in these small patients, the potential for destabilization of the construct, or planned future removal of the implants once the fracture healed. When fluoroscopy is not available, particular care should be taken to evaluate the placement of the proximal screw on postoperative radiographs. If violation of the proximal tibial physis or the articular surface is apparent, revision should be undertaken prior to recovering the patient.

Intraarticular screw placement causes chondral damage, exacerbates chondrolysis and osteoarthritis, and therefore should be avoided [17]. In the two cases with penetration of the proximal screw into the stifle joint, both patients recovered well and did not have any clinical lameness at the time of last follow-up. It is possible that the lack of apparent pain or lameness in these cases is due to skiving, which is defined in human literature as the condition when the subchondral plate is disrupted while the underlying cartilage is physically displaced without the screw entering the joint [18]. CT scan has a greater sensitivity compared to radiography when diagnosing skiving, which was not performed in any of the cases in this study [18]. It is possible that these cases had skiving as opposed to intraarticular screw violation, which accounts for the favorable outcome in both patients.

One case was managed with an intramedullary pin and type 1a external fixator (Fig 5) and recovered well without any noted complications. This type of fracture fixation method may be useful for PTMF given the limited bone stock of the proximal fracture fragment, its proximity to the proximal tibial physis and the potential for rapid healing due to patient age [19].

Of particular concern in canine PTMF is the cranial displacement of the distal segment, which results in caudal tipping of the proximal tibia and risks the development of an excessive tibial plateau angle, potentially increasing strain on the cranial cruciate ligament (Figs 2 and 7) [10]. Surgical intervention allows for more accurate reduction of the fracture fragments, therefore reducing the risk of caudal tipping of the proximal segment. None of the patients in this study developed cranial cruciate ligament rupture, but this may be attributed to the short-term follow-up. We also recognized that PTMF configuration may result in distal tibial valgus. This may also be corrected by the superior reduction and alignment afforded by surgical intervention as opposed to medical management with external coaptation.

Limitations of this study include those inherent to its retrospective nature, a low number of cases that precluded statistical analysis between and within the various groups, inconsistent methods of surgical stabilization employed, inconsistent use of postoperative bandages and splints, and a lack of consistency of radiographic evaluation among the cases reviewed. Further studies are needed to determine the most effective method of surgical intervention for fracture repair.

Despite limitations within this study, we reported surgical and medical management of PTMF fractures along with the short-term outcome. Subjectively, surgical management has a more predictable outcome and can prevent conformational changes to the proximal tibia that may predispose patients to cranial cruciate ligament rupture and angular limb deformity.

## References

[1] Seamon J.A. and Simpson A.M. Tibial fractures. Clin Tech Small Anim Pract. 2005; 19: 151–167 10.1053/j.ctsap.2004.09.007 15712461

[2] Gorse M.J. Using external skeletal fixation for fractures of the radius and ulna and tibia. Vet Med 1998; 93: 463–467

[3] Marretta S.M. and Schrader S.C. Physeal injuries in the dog: a review of 135 cases. JAVMA 1983; 182: 708–710 6841256

[4] Schmokel H. Weber U. and Hartmeier G. Salter-II fracture of the proximal tibia with avulsion of the tuberositas tibiae in the dog. Schwizer Archiv fur Tierheilkunde 1995; 137: 124–128 7660096

[5] Pratt J.N. Avulsion of the tibial tuberosity with separation of the proximal tibial physis in seven dogs. Vet Rec 2001; 149; 352–356 doi: 10.1136/vr.149.12.352 11594381

[6] Boone E.G., Johnson A.L., Hohn R.B. Distal tibial fractures in dogs and cats. J Am Vet Med Assoc 1986; 188: 36–40. 3944006

[7] Boone E.G., Johnson A.L., Montavon P, et al. Fractures of the tibial physis in dogs and cats. J Am Vet Med Assoc 1986; 188: 41–45. 3944007

[8] Unger M., Montavon P.M., Heim U.F.A. Classification of fractures of long bones in the dog and cat: introduction and clinical application. Vet Comp Orthop Traumatol 1990; 3: 41–50.

[9] Deahl L., Ben-Amotz R., Caceres A.V., and Agnello K.A. Proximal metaphyseal fractures in immature dogs. Vet Comp Orthop Traumatol 2017: 1–6 28393960 10.3415/VCOT-16-11-0154

[10] Haynes KH., Biskup J., Freeman A., et al. Effect of tibial plateau angle on cranial cruciate ligament strain: an ex vivo study in the dog. Vet Surg 2015: 44: 46–49 doi: 10.1111/j.1532-950X.2014.12219.x 24902869

[11] Tobias K. and Johnston S.A. Small Animal Veterinary Surgery. Second Edition. St. Louis, MO: Elsevier/Saunders, 2016. Print

[12] Meeson RL, Davidson C, Arthurs GI. Soft-tissue injuries associated with cast application for distal limb orthopaedic conditions. Vet Comp Orthop Traumatol 2011;24:126–131 doi: 10.3415/VCOT-10-03-0033 21225085

[13] Boekhout-Ta C.L., Kim S.E., Cross A.R., Evans R., Pozzi A. Closed reduction and fluoroscopic-assisted percutaneous pinning of 42 physeal fractures in 37 dogs and 4 cats. Vet Surg 2017; 46: 103–110. doi: 10.1111/vsu.12582 27925240

[14] Saglam M. and Kaya U. Treatment of proximal tibial fractures by cross pin fixation in dogs. Turk J Vet Anim Sci 2004; 28: 799–805.

[15] Newman M.E. and Milton J.L. Closed reduction and blind pinning of 29 femoral and tibial fractures in 27 dog and cats. J Am Anim Hosp Assoc. 1989; 25: 61–68.

[16] Kennon J.C., Ganey T.M., Glenn Glaston R., Ogden J.A. J Pediatr Orthop 2013; 33: 857–861. doi: 10.1097/BPO.0b013e31829c008b 23812151

[17] Ceylan H.H., Erden T., Kapicioglu M., Küçükdurmax F. A novel method to assess intraarticular screw penetration into joint surface. Jt Dis Relat Surg 2020: 31 (2): 218–222. doi: 10.5606/ehc.2020.71764 32584717 PMC7489154

[18] Takemoto R.C., Gage M.J., Rybak L, Walsh M., Egol K.A. Articular cartilage skiving: the concept defined. Journal of Hand Surgery (European Volume).2011. 36 (E) 5. 364–369. doi: 10.1177/1753193411398196 21372050

[19] Aronsohn M.G., Burk R.L. Unilateral Uniplanar External Skeletal Fixation for Isolated Diaphyseal Tibial Fractures in Skeletally Immature Dogs. Vet Surg 2009; 38: 654–658. doi: 10.1111/j.1532-950X.2009.00553.x 19573070

