# Supplementary material for: Surgical and medical management in the treatment of proximal tibial metaphyseal fracture in immature dogs
Source: PLoS One. 2022 Jun 2;17(6):e0268378. doi: 10.1371/journal.pone.0268378 (PMC9162371; doi:10.1371/journal.pone.0268378)
Supplement: Attachment — Submitted filename: Response to Reviewers.docx [file pone.0268378.s001.docx]

Ron Ben-Amotz, DVM, DACVS, DECVS, CCRP

Section Head, Small Animal Orthopedics

Koret School of Veterinary Medicine

Lecturer, The Hebrew University of Jerusalem

P.O. Box 12, Rehovot, 76100

March 22, 2022

To the Editors of PLOS ONE and Reviewer of “Surgical and medical management in the treatment of proximal tibial metaphyseal fracture in immature dogs”:

Thank you very much for your consideration of this research article. Below is an explanation of changes made in order to appropriately satisfy the PLOS ONE publication criteria:

Thank you for your comment regarding the description in the Methods section. We have provided additional details regarding the data sources used and these changes are noted within the manuscript. The change in the dates reflects when the cases initially presented to the hospitals. We have also included information regarding the number of hospitals from which data was sourced as well as their respective locations.

We have appropriately addressed PLOS ONE’s publication criteria regarding data availability within the portal. At the time of the initial submission, a misunderstanding prompted the “N/A” response. This has now been resolved.

One of the references was accidentally duplicated in the reference list twice, and this has now been corrected in the manuscript. One of the references was incorrectly cited in the introduction, so this was corrected.

Thank you for the reviewer comment: “One suggestion, which is not required, is to incorporate some aspect of 'time' into the assessment. That is, it would be beneficial for the reader to have some sense of the duration of the various interventions, the time between various interventions and/or the time at which the fracture was considered 'healed'”. Unfortunately, the timing data requested is not available for all of the cases we included in this study. The authors were concerned that the inclusion of incomplete data would detract from the study and potentially confuse the readers, particularly since multiple treatment modalities were described.

Please let us know if any further changes are needed to meet the criteria of PLOS ONE. We appreciate your time, consideration and further feedback you may provide.

Sincerely,

Ron Ben-Amotz, Carly Sullivan, Joshua Zuckerman, Daniel James, Karl Maritato, Emily Morrison, and Riccarda Schuenemann
